# Evaluation of the Valve Academic Research Consortium-2 Criteria for Myocardial Infarction in Transcatheter Aortic Valve Implantation: A Prospective Observational Study

**DOI:** 10.1371/journal.pone.0130423

**Published:** 2015-06-12

**Authors:** Lennart Nilsson, Carl-Fredrik Appel, Henrik Hultkvist, Farkas Vánky

**Affiliations:** 1 Department of Cardiology, University Hospital, Linköping, Sweden; 2 Department of Thoracic and Vascular Surgery, University Hospital, Linköping, Sweden; 3 Department of Medical and Health Sciences, Linköping University, Linköping, Sweden; Harvard Medical School, UNITED STATES

## Abstract

**Objective:**

To evaluate the relevance of the individual components of the Valve Academic Research Consortium (VARC)-2 criteria for periprocedural myocardial infarction (MI) in transcatheter aortic valve implantation (TAVI). The association between biomarkers and adverse procedural outcome has been established. However, the additive prognostic importance of signs and symptoms are more uncertain.

**Methods:**

A total of 125 consecutive TAVI patients were prospectively included in this study. Biomarkers for MI were analyzed and signs and symptoms according to VARC-2 criteria were collected from clinical records.

**Results:**

The criteria of elevated biomarkers and of signs or symptoms were found in 27 (22%) and 32 (26%) of the patients, respectively. According to VARC-2 definition, 12 (10%) had MI. VARC-2 definition of MI, Troponin T (TnT) > 600 ng/L, and presence of signs or symptoms correlated with 6 months mortality, prolonged ICU stay, elevation of N-terminal prohormone brain natriuretic peptide, and renal impairment. No signs or symptoms were found in 7 (44%) of the patients who fulfilled the criterion of elevated TnT > 600 ng/L. In the group with positive TnT criterion, there were no significant differences between those with and without signs or symptoms in respect to levels of TnT (1014 [585–1720] ng/L versus 704 [515–905] ng/L, p = 0.17) or creatine kinase-MB (36 [25–52] μg/L versus 29 [25–39] μg/L, p = 0.32). In the multivariate Cox regression analysis, TnT > 600 ng/L was the only significant independent variable associated with 6-months postprocedural mortality.

**Conclusions:**

Myocardial injury in TAVI, measured with biomarkers, correlates well with adverse procedural outcome. In this study it is also the strongest predictor for early postprocedural mortality. The additional requirement of signs or symptoms for the diagnosis of MI results in omission of a considerable number of clinically significant MI.

## Introduction

Since the first percutaneous transcatheter aortic valve implantation (TAVI) procedure in human in 2002 described by Cribier et al., TAVI techniques have developed to provide an alternative approach to surgical aortic valve replacement primarily in patients with high comorbidity and high surgical risk [1,2]. The TAVI procedure is under development and in recent years, it has been provided to patient groups with lower surgical risk [3].

There are needs of standardized endpoint definitions, to obtain comparable data on periprocedural outcomes of cardiac interventions and for continuous evaluations. The definitions must be easy to interpret, be applicable in the clinical setting and preferably be of clinical and prognostic relevance. The Valve Academic Research Consortium (VARC) has published endpoint definitions for TAVI procedures.[4] The VARC definitions have been used in publications on TAVI and thereby facilitate the comparisons between different studies.

The updated VARC-2 definition for periprocedural myocardial infarction (MI; <72 h after the index procedure) requires fulfillment of criteria for both biomarkers of myocardial injury and signs or symptoms. The complete definition is: new ischemic symptoms (e.g., chest pain or shortness of breath), or new ischemic signs (e.g., ventricular arrhythmias, new or worsening heart failure, new ST-segment changes, hemodynamic instability, new pathological Q-waves in at least 2 contiguous leads, imaging evidence of new loss of viable myocardium or new wall motion abnormality) and elevated cardiac biomarkers (preferable creatine kinase-MB [CK-MB]) within 72 h after the index procedure, consisting of at least 1 sample postprocedure with a peak value exceeding 15x as the upper reference limit for troponin or 5x for CK-MB. If cardiac biomarkers are increased at baseline (>99th percentile), a further increase of at least 50% postprocedure is required and the peak value must exceed the previously stated limit [4].

Elevation of biomarkers of myocardial injury is an inevitable criterion for the diagnosis of MI. However, it is recognized that the TAVI procedure itself may result in an elevation of biomarkers and therefore the levels of CK-MB and troponin are set to 5 and 15 times the upper reference limit, respectively, for the diagnosis of periprocedural MI [4].

Signs and symptoms, such as chest pain, shortness of breath, electrocardiogram (ECG) changes and echocardiographic findings might be influenced by the TAVI procedure itself and therefore possibly less specific to TAVI-related periprocedural MI as they are to spontaneous acute MI. Furthermore, many patients are sedated and are unable to adequately provide symptoms. The objective of this study was to evaluate the relevance of the individual components in the VARC-2 criteria for periprocedural MI in TAVI. To our knowledge, the impact of signs and symptoms on the diagnosis of MI in TAVI or on the procedural outcome has not previously been evaluated.

## Methods

In order to evaluate different aspects of the TAVI procedure, consecutive patients, scheduled for TAVI, were prospectively included in the study after informed, written consent. The study was approved by the Regional Ethical Review Board in Linköping, Sweden (M 198–07, T 26–8) and conducted in accordance with the Helsinki declaration.

TAVI, as treatment for symptomatic aortic stenosis, was primarily considered in case of high surgical risk in terms of predicted mortality. Patients with moderate or low surgical risk scores were also accepted for TAVI, in case of specific risk factors for surgical aortic valve replacement. Such specific risk factors were heavily calcified ascending aorta, previous coronary surgery with patent grafts, age > 90 years or otherwise fragile patient, sequelae of chest radiotherapy, immunosuppressive therapy, and malignancy scheduled for surgery, cold agglutinin disease, and highly aggravated pulmonary or renal dysfunction. Edwards Sapien valve bioprostheses (Edwards Lifesciences Inc, Irvine, Calif) were used for implantation in all procedures. The procedure has been previously described in detail [5].

During the study period from the 1^st^ of September 2009 to the 1^st^ of February 2013, 144 TAVI procedures were conducted at our center and 125 patients accepted study inclusion. The transfemoral (TF) and the transapical (TA) approaches were used in 88 and 37 patients, respectively. Doppler echocardiography was performed the day before and 3–4 days after the TAVI intervention. In addition to our clinical routine, blood samples were collected before procedure for N-terminal prohormone brain natriuretic peptide (NT-proBNP), Creatinine, and Cystatin C, the first day after procedure for NT-proBNP and CK-MB, and the third day after procedure for NT-proBNP, Creatinine, Cystatin C, and Troponin T (TnT). The upper reference limit for high sensitive TnT and CK-MB was 15 ng/L and 5 μg/L, respectively. The times for CK-MB and TnT measurements were chosen upon the peak times of these biomarkers after TAVI described by Rodés-Cabau [6].

Data, specific for this study, were recorded in a clinical research form and other clinical data were registered in, and obtained from the clinical database, Carath (Fujitsu Ltd, Tokyo, Japan). Collection of data on signs and symptoms of periprocedural MI according to VARC-2 criteria were registered without knowledge of biochemical markers for myocardial injury or ECG. Signs and symptoms were registered from the end of the procedure to 72 hours postprocedurally. Echocardiography and ECG were separately evaluated by cardiologists blinded for all other patient data. Mortality data were retrieved from the Swedish Civil Registry.

### Statistical methods

Categorical variables are presented as numbers (percent) and continuous variables as mean (± SD) or median (25^th^ to 75^th^ percentile) for non-Gaussian distributed data. Nonparametric tests were performed in all group comparisons. Fisher’s exact test was used for categorical variables and Mann-Whitney-U test was used for continuous variables. Spearman correlations were used to assess associations between variables. Cox regression analyses were used to determine the univariate association between the different variables and 6-months mortality. Continuous variables were also tested in a categorized form. Collinearity diagnostics were performed and no signs of multicollinearity were found. A multivariate forward stepwise Cox regression analysis was then performed including variables with p value < 0.15 in the univariate analysis. Sensitivity and specificity were calculated for TnT >600 ng/L, signs and symptoms, and the VARC-2 criteria for MI in relation to 6-months mortality. Receiver-operating characteristic curve analysis was performed for TnT and CK-MB. A p-value <0.05 was considered statistically significant. All statistical analyses were performed using IBM SPSS Statistics for Windows, Version 21.0 (IBM Corporation, Armonk, NY, USA).

## Results

The baseline characteristics and procedural data for the entire study population and the TA and TF groups are shown in Table 1. In the TA group Euroscore II was higher and peripheral artery disease was more frequent than in the TF group. No significant differences were found in the procedural data between the groups.

**Table 1:** Baseline patient characteristics and procedural data.

	All n = 125	TA n = 37	TF n = 88	p-value
***Patient characteristics***				
Age (years)	80 ± 8	81 ± 7	80±8	0.60
Female gender, n (%)	65 (52)	17 (46)	48 (55)	0.44
EuroSCORE II (%)	6.1 ± 4.6	8.3 ± 6.0	5.2 ± 3.6	0.002
Body mass index (kg/m^2^)	25 ± 4	24 ± 3	26 ± 4	0.10
Diabetes mellitus, n (%)	23 (18)	6 (16)	17 (19)	0.80
COPD, n (%)	15 (12)	5 (14)	10 (11)	0.77
Cerebrovascular disease, n (%)	20 (16)	5 (14)	15 (17)	0.79
Peripheral artery disease, n (%)	23 (18)	16 (43)	7 (8)	<0.001
Hypertension, n (%)	77 (62)	20 (54)	57 (65)	0.32
Angina pectoris, n (%)	24 (19)	4 (11)	20 (23)	0.14
Previous myocardial infarction, n (%)	32 (26)	11 (30)	21 (24)	0.57
Previous PCI, n (%)	42 (34)	14 (38)	28 (32)	0.54
Previous cardiac surgery, n (%)	34 (27)	12 (32)	22 (25)	0.39
Congestive heart failure, n (%)	53 (42)	17 (46)	36 (41)	0.69
NYHA functional class, n (%)				
II	8 (6)	1 (3)	7 (8)	0.43
III	107 (86)	31 (84)	76 (86)	0.78
IV	9 (7)	5 (14)	4 (5)	0.12
Hemoglobin (g/L)	131 ± 15	128 ± 15	132 ± 15	0.19
Plasma Cystatin C (mg/L)	1.63 ± 0.54	1.69 ± 0.52	1.60 ± 0.55	0.27
Plasma Creatinine (μmol/L	110 ± 50	115 ± 58	109 ± 47	0.78
NT-proBNP (ng/L)	2070 (1110–4960)	2875 (1775–5905)	1825 (820–4750)	0.03
***Preoperative echocardiography***				
LVEF 40–49%	25 (20)	9 (24)	16 (18)	0.47
LVEF 30–39%	16 (13)	5 (14)	11 (13)	1.00
LVEF <30%	5 (4)	3 (8)	2 (2)	0.15
Systolic PAP >60 mmHg, n (%)	28 (22)	11 (30)	17 (20)	0.24
Maximal aortic transvalvular velocity (m/s)	4.5 ± 0.7	4.7 ± 0.7	4.5 ± 0.7	0.21
Mean aortic valve gradient (mmHg)	53 ± 18	54 ± 19	52 ± 18	0.59
Aortic valve area (cm^2^)	0.56 ± 0.16	0.52 ± 0.15	0.58 ± 0.17	0.05
***Procedural data***				
Procedure time (min)	107 ± 45	107 ± 37	107 ± 48	0.24
Rapid pacing runs	2 ± 1	2 ± 1	2 ± 1	0.45
Use of ECC, n (%)	5 (4)	3 (8)	2 (2)	0.15
Use of contrast, n (%)	101 (81)	31 (83)	70 (80)	0.80
Contrast amount, when used (mL)	50 (25–75)	50 (25–85)	50 (30–69)	0.47
Prosthesis size				
23 mm	49 (39)	13 (35)	36 (41)	0.69
26 mm	68 (54)	21 (57)	47 (53)	0.84
29 mm	8 (6)	3 (8)	5 (6)	0.69

Data are presented as numbers (%), mean ± SD or median (25th to 75th percentile). COPD = chronic obstructive pulmonary disease; ECC = extracorporeal circulation; LVEF = Left ventricular ejection fraction; NYHA = New York Heart Association class; NT-proBNP = N-terminal prohormone brain natriuretic peptide; PCI = percutaneous coronary intervention; PAP = pulmonary artery pressure; TA = transapical approach; TF = transfemoral approach.

Mortality and postprocedural in-hospital outcome measures are listed in Table 2. The TA group had significantly increased need of respirator treatment after the procedure as well as longer ICU-stay compared to the TF group.

**Table 2:** Mortality and postprocedural in-hospital outcome measures.

	All n = 125	TA n = 37	TF n = 88	p-value
30-day mortality, n (%)	9 (7)	5 (14)	4 (5)	0.12
6-months mortality, n (%)	15 (12)	8 (22)	7 (8)	0.07
Plasma CK-MB, day 1 (μg/L)	10 (6–21)	20 (12–29)	7 (4–16)	<0.001
Plasma TroponinT, day 3 (ng/L)	185 (90–422)	422 (250–818)	127 (71–293)	<0.001
Plasma Cystatin C day 3 (mg/L)	1.63 (1.33–1.98)	1.68 (1.35–1.29)	1.57 (1.29–1.87)	0.24
Plasma Creatinine day 3 (μmol/L)	88 (71–114)	107 (76–142)	84 (68–106)	0.06
Plasma Creatinine elevation >50%, n (%)	4 (3)	3 (10)	1 (1)	0.07
NT-proBNP day 1 (ng/L)	3340 (1710–5320)	4710 (3340–8810)	3200 (1370–4490)	0.002
NT-proBNP day 3 (ng/L)	3035 (1345–5985)	4830 (3080–7280)	2290 (1020–5050)	<0.001
Hemodialysis	3 (2)	3 (8)	0 (0)	0.03
Postop atrial fibrillation/flutter	17 (14)	7 (22)	10 (13)	0.25
Stroke, n (%)	5 (4)	1 (3)	4 (5)	1.00
Erythrocyte transfusion, n (%)	50 (40)	22 (59)	28 (32)	0.005
Erythrocyte transfusion* (mL)	863 (300–1475)	875 (550–1375)	850 (300–1913)	0.94
Plasma transfusion, n (%)	50 (40)	21 (57)	29 (33)	0.02
Plasma transfusion* (mL)	600 (600–1475)	875 (600–1475)	600 (600–1400)	1.00
Thrombocyte transfusion, n (%)	8 (6)	3 (8)	5 (6)	0.69
Thrombocyte transfusion* (mL)	288 (263–450)	300 (250–500)	275 (275–400)	0.88
Patients to ICU, n (%)	112 (90)	36 (97)	76 (86)	0.11
ICU-stay (h)	20 (17–23)	23 (18–35)	19 (16–23)	0.004
Patients on ventilator after procedure, n (%)	61 (49)	29 (78)	32 (36)	<0.001
Time on ventilator (h)	3 (2–5)	4 (2–9)	3 (2–4)	0.14

Data are presented as numbers (%) or median (25th to 75th percentile). CK-MB = creatine kinase-muscle, brain isotype; ICU = intensive care unit; NT-proBNP = N-terminal prohormone brain natriuretic peptide.

* Average volume given to patients who received transfusion.

### Biomarkers of myocardial injury

Both the median TnT level at 72 hours and the CK-MB level at 24 hours postprocedurally were higher in the TA group compared to the TF group (Table 2). In the total study population, there was a positive correlation between TnT and CK-MB (r = 0.72, p<0.001). Sixteen patients (13%) had TnT > 600 ng/L, 18 patients (14%) had CK-MB > 25 μg/L, and 27 patients (22%) had TnT > 600 ng/L or CK-MB > 25 μg/L postprocedurally. Nine of the patients with TnT > 600 ng/L had CK-MB < 25 μg/L. Two patients in the TF group had CK-MB > 40 μg/L without TnT elevation above 250 ng/L, both with lower limb ischemia requiring fasciotomy.

### Electrocardiographic findings

Prior to the procedure, only 27 patients (22%) had a normal ECG, whereas 90 patients (72%) had a pathological ECG and in 8 patients (6%) the ECG was not interpretable due to ventricular pacing. Typical ECG findings were Q-waves (n = 11 [9%]), ST-depressions (n = 45 [36%]) and bundle branch or fascicular block (n = 27 [22%]).

After the procedure, 8 patients (6%) had developed ECG changes according to the VARC-2 criteria: 6 showed new ST-depressions and 2 had pathological Q-waves. Additionally, 7 patients (6%) developed a left bundle branch block and 3 patients (2%) a left anterior fascicular block.

### Signs and symptoms according to the VARC-2 MI definition

Fourteen patients (38%) in the TA group and 18 patients (20%) in the TF group (p = 0.07) had any sign or symptom. Echocardiographic signs of myocardial injury were only found in the TA group, in 5 (14%) of the patients. The postprocedural levels of TnT and CK-MB for patients with and without symptoms or signs are presented in Table 3. Seven (44%) of the patients with TnT > 600 ng/L and 9 (50%) of the patients with CK-MB>25 μg/L had no signs and symptoms of MI. In patients with TnT > 600 ng/L, there were no significant differences between those with and those without signs and symptoms in respect to TnT or CK-MB levels.

**Table 3:** Markers of myocardial injury in patients with and without signs and symptoms.

	Signs or symptoms	Signs or symptoms	p-value
All patients (n = 125)	Yes (n = 32)	No (n = 93)	
TnT day 3 (ng/L)	478 (190–871)	140 (80–350)	<0.001
TnT day 3 > 225 ng/L, n (%)	20/28 (71)	31/87 (36)	0.001
TnT day 3 > 600 ng/L, n (%)	9/28 (32)	7/87 (8)	0.003
CK-MB day 1 (μg/L)	18 (8–29)	9 (5–20)	0.008
CK-MB day 1 > 25 μg/L, n (%)	9/29 (31)	9/74 (12)	0.04
Patients with TnT > 600 ng/L (n = 16)	Yes (n = 9)	No (n = 7)	
TnT day 3 (ng/L)	1400 (988–1940)	860 (720–1000)	0.14
CK-MB day 1 (μg/L)	38 (20–54)	23 (8–25)	0.22
CK-MB day 1 > 25 μg/L	6/9 (67)	1/6 (17)	0.06
Patients with TnT > 600 ng/L or CK-MB > 25 μg/L (n = 26)	Yes (n = 12)	No (n = 14)	
TnT day 3 (ng/L)	1014 (585–1720)	704 (515–905)	0.17
CK-MB day 1 (μg/L)	36 (25–52)	29 (24–39)	0.32
CK-MB day 1 > 25 μg/L, n (%)	9/12 (75)	9/14 (64)	0.68

Data are presented as numbers (%) or median (25th to 75th percentile). CK-MB = creatine kinase-muscle, brain isotype; TnT = Troponin T.

The postprocedural outcome in terms of mortality, NT-proBNP, renal function, and duration of ICU stay for the study population divided upon levels of biomarkers of MI, presence of signs and symptoms, and MI according to the VARC-2 definition are shown in Table 4.

**Table 4:** Markers of myocardial injury, signs or symptoms, and VARC-2 criteria of periprocedural MI in relation to mortality, changes in NT-proBNP, cystatin C and creatinine, and duration of ICU-stay.

	30-day mortality	p Value	6-months mortality	p Value	NT-proBNP ΔDay 0–3	p Value	CystatinC Δ Day 0–3	p Value	Creatinine elevation >50%	p Value	ICU time (h)	p Value
**TnT day 3**												
>600ng/L	4 (25%)	0.007	6 (38%)	0.003	3470 (100–8630)	0.002	0.36 (0.06–0.57)	0.01	3 (21%)	0.009	32 (20–93)	<0.001
<600ng/L	3 (3%)		7 (7%)		390 (-510-1770)		0.03 (-0.06–0.18)		1 (1%)		19 (17–23)	
**CK-MB day1**												
>25μg/L	3 (17%)	0.19	3 (17%)	0.7	1440 (10–2370)	<0.001	0.23 (0.02–0.53)	0.02	3 (17%)	0.02	23 (18–71)	0.17
<25μg/L	6 (7%)		10 (12%)		270 (-1220-1570)		0.04 (-0.09–0.18)		1 (1%)		20 (18–23)	
**Symptoms or signs**												
Yes (n = 32)	8 (25%)	<0.001	9 (28%)	0.003	1710 (440–4750)	<0.001	0.25 (0.04–0.45)	0.005	4/27 (15%)	0.003	32 (20–93)	<0.001
No (n = 93)	1 (1%)		6 (7%)		45 (-1235-1175)		0.02 (-0.07–0.17)		0		19 (17–23)	
**TnT>600 ng/L and symptoms or signs**												
Yes (n = 9)	4 (44%)	0.09	5 (56%)	0.15	2300 (990–11620)	0.83	0.49 (0.36–0.67)	0.11	3/8 (38%)	0.21	74 (67–95)	0.006
No (n = 7)	0		1 (13%)		6180 (1440–6230)		0.11 (-0.09–0.32)		0		20 (17–32)	
**VARC2 criteria MI**												
Yes (n = 12)	4 (33%)	0.005	5 (42%)	0.006	2230 (1160–14970)	0.002	0.45 (0.21–0.78)	<0.001	4/11 (36%)	0.001	67 (23–95)	<0.001
No (n = 113)	5 (4%)		10 (9%)		90 (-1195-1460)		0.03 (-0.06–0.18)		0		19 (17–23)	

Data are presented as numbers (%) or median (25th to 75th percentile). CK-MB = creatine kinase-muscle, brain isotype; ICU = intensive care unit; MI = myocardial infarction; NT-proBNP = N-terminal prohormone brain natriuretic peptide; TnT = troponin T; VARC = Valve Academic Research Consortium.

### The impact of myocardial injury on 6-months mortality

Baseline patient characteristics and procedural data according to the cumulative all-cause mortality at 6-months follow-up are shown in Table 5. The results of the univariate Cox regression analysis of risk factors for 6-months cumulative mortality are presented in Table 6.

**Table 5:** Baseline patient characteristics and procedural data according to the cumulative all-cause mortality at 6-months follow-up.

Cumulative mortality:	No (n = 110)	Yes (n = 15)	p-value
***Patient characteristics***			
Age (years)	80±8	81±10	0.64
Female gender, n (%)	57 (52)	8 (53)	1.0
EuroSCORE II (%)	6.3 ± 4.8	5.3 ± 2.7	0.85
Body mass index (kg/m^2^)	25 ± 4	24±3	0.68
Diabetes mellitus, n (%)	18 (16)	5 (33)	0.15
COPD, n (%)	14 (13)	1 (7)	0.69
Cerebrovascular disease, n (%)	17 (15)	3 (20)	0.71
Peripheral artery disease, n (%)	20 (18)	3 (20)	1.0
Hypertension, n (%)	69/103 (67)	8/13 (62)	0.76
Angina pectoris, n (%)	21 (19)	3 (20)	1.0
Previous myocardial infarction, n (%)	29 (26)	3 (20)	0.76
Previous PCI, n (%)	38/109 (35)	4 (27)	0.77
Previous cardiac surgery, n (%)	32 (29)	2 (13)	0.35
Congestive heart failure, n (%)	44 (40)	9 (60)	0.17
NYHA functional class, n (%)			
II	8 (7)	0	0.60
III	93 (85)	14 (93)	0.69
IV	8 (7)	1 (7)	1.0
Hemoglobin (g/L)	131 ± 15	128 ± 17	0.23
Plasma Cystatin C (mg/L)	1.61 ± 0.52	1.75 ± 0.71	0.46
Plasma Creatinine (μmol/L	109 ± 46	123 ± 77	0.96
NT-proBNP (ng/L)	2030 (940–5440)	2250 (1380–4020)	0.87
***Preoperative echocardiography***			
LVEF 40–49%	18 (16)	7 (47)	0.01
LVEF 30–39%	16 (15)	1 (7)	0.69
LVEF <30%	5 (5)	0	1.0
Systolic PAP >60 mmHg, n (%)	27 (25)	1 (7)	0.19
Maximal aortic transvalvular velocity (m/s)	4.5 ± 0.7	4.6 ± 0.8	0.96
Mean aortic valve gradient (mmHg)	53 ± 18	52 ± 21	0.78
Aortic valve area (cm^2^)	0.56 ± 0.16	0.55 ± 0.18	0.62
***Procedural data***			
Transapical approach	29 (26)	8 (53)	0.07
Procedure time (min)	106 ± 45	118 ± 44	0.28
Rapid pacing runs	2 ± 1	2 ± 1	0.73
Use of ECC, n (%)	4 (4)	1 (7)	0.48
Use of contrast, n (%)	88 (80)	17 (87)	0.73
Contrast amount, when used (mL)	50 (25–75)	50 (35–75)	0.58
Prosthesis size			
23 mm	43 (39)	6 (40)	1.0
26 mm	59 (54)	9 (60)	0.78
29 mm	8 (7)	0	0.59
Plasma CK-MB, day 1 (μg/L)	10 (6–21)	16 (8–23)	0.36
Plasma TnT, day 3 (ng/L)	157 (90–400)	469 (176–1500)	0.03
TnT day 3 > 600 ng/L, n (%)	10/102 (10)	6/13 (46)	0.003
Signs or symptoms n (%)	23 (21)	9 (60)	0.003
Patients with TnT > 600 ng/L or CK-MB > 25 μg/L	21 (19)	6 (40)	0.09
VARC2 criterion for MI, n (%)	7 (6)	5 (33)	0.006
Need for hemodialysis	1 (1)	2 (13)	0.04
Stroke within 30 days	5 (5)	0	1.0

Data are presented as numbers (%), mean ± SD or median (25th to 75th percentile). COPD = chronic obstructive pulmonary disease; ECC = extracorporeal circulation; LVEF = Left ventricular ejection fraction; MI = myocardial injury; NYHA = New York Heart Association class; NT-proBNP = N-terminal prohormone brain natriuretic peptide; PCI = percutaneous coronary intervention; PAP = pulmonary artery pressure; CK-MB = creatine kinase-muscle, brain isotype; TnT = Troponin T.

**Table 6:** Univariate Cox regression analysis of risk factors for 6-months cumulative mortality.

Variable	n (%) of patients	6 months mortality n (%)	HR	CI (95%)	p-value
Patients	125	15 (12%)			
Age (years)					
Mean: 80			1.005	0.94–1.07	0.88
Sex					
Male	55 (48)	7 (13)	1.00		
Female	65 (52)	8 (12)	1.10	0.40–3.04	0.85
Body mass index (kg/m2)					
Mean: 25			0.95	0.82–1.10	0.51
Diabetes (insulin or orally treated)					
No	102 (82)	10 (10)	1.00		
Yes	23 (18)	5 (22)	2.26	0.77–6.62	0.14
Hypertension					
No	48 (38)	7 (15)	1.00		
Yes	77 (62)	8 (10)	0.82	0.27–2.51	0.73
Cerebrovascular disease					
No	105 (84)	12 (11)	1.00		
Yes	20 (16)	3 (2)	1.33	0.37–4.70	0.66
Previous MI					
No	93 (74)	12 (13)	1.00		
Yes	32 (26)	3 (9)	0.69	0.20–2.46	0.57
Congestive heart failure					
No	72 (58)	6 (8)	1.00		
Yes	53 (42)	9 (17)	2.15	0.77–6.04	0.15
Peripheral artery disease					
No	102 (82)	12 (12)	1.00		
Yes	23 (18)	3 (13)	1.14	0.32–4.05	0.84
Hemoglobin (g/L)					
Mean: 131			0.98	0.95–1.02	0.33
Plasma Cystatin C (mg/L)					
Mean: 1.63			1.57	0.67–3.68	0.30
Plasma Creatinine (μmol/L)					
Mean: 110			1.005	0.99–1.01	0.27
Creatinine clearence (mL/minute)					
≥50	50 (40)	6 (12)	1.00		
<50	75 (60)	9 (12)	1.026	0.37–2.88	0.96
Mean: 51			0.997	0.98–1.02	0.77
NYHA class					
I-III	116 (93)	14 (12)	1.00		
IV	9 (7)	1 (11)	0.97	0.13–7.40	0.98
EuroSCORE II					
Mean: 6.1			0.95	0.83–1.09	0.45
Systolic PAP (mmHg)					
≤60	97 (78)	14 (14)	1.00		
>60	28 (22)	1 (4)	0.23	0.03–1.77	0.16
Mean: 49			0.96	0.91–1.01	0.14
LV dysfunktion moderate or severe					
No	103 (82)	14 (14)	1.00		
Yes	22 (18)	1 (5)	0.31	0.04–2.38	0.26
Approach					
Transfemoral	88 (70)	7 (8)	1.00		
Transapical	37 (30)	8 (22)	2.98	1.08–8.23	0.04
Procedure time (min)					
Mean: 107			1.005	0.99–1.01	0.29
Rapid pacing runs					
Mean: 2.0			0.83	0.46–1.47	0.51
Plasma CK-MB, day 1 (μg/L)					
≤225	85 (83)	12 (14)	1.00		
>225	18 (17)	3 (17)	1.50	0.41–5.47	0.54
Median: 10			1.03	1.01–1.05	0.007
Plasma TroponinT, day 3 (ng/L)					
≤600	99 (86)	9 (9)	1.00		
>600	16 (14)	6 (38)	6.68	2.24–19.9	0.001
Median: 185			1.001	1.001–1.002	0.001
Signs or symptoms					
No	93 (74)	6 (6)	1.00		
Yes	32 (26)	9 (28)	5.29	1.88–14.9	0.003
TnT > 600 ng/L or CK-MB > 225 μg/L					
No	98 (78)	9 (9)	1.00		
Yes	27 (22)	6 (22)	2.62	0.93–7.37	0.07
VARC2 criterion for MI					
No	113 (90)	10 (9)	1.00		
Yes	12 (10)	5 (42)	6.01	2.05–17.7	0.002
Stroke within 30 days					
No	110 (96)	15 (14)	1.00		
Yes	5 (4)	0	0.047	0.00–6110	0.61
Need for hemodialysis					
No	122 (98)	13 (11)	1.00		
Yes	3 (2)	2 (67)	12.2	2.70–55.2	0.03

For continuous variables hazard ratios are calculated per unit change. CI = confidence interval; ECC = extracorporeal circulation; HR = hazard ratio; LV = Left ventricular; MI = myocardial injury; NYHA = New York Heart Association class; NT-proBNP = N-terminal prohormone brain natriuretic peptide; PCI = percutaneous coronary intervention; PAP = pulmonary artery pressure; CK-MB = creatine kinase-muscle, brain isotype; TnT = Troponin T.

Variables with p < 0.15 were tested in a forward stepwise way in a multivariate Cox regression model. Collinearity diagnostics were performed and no signs of multicollinearity were found. In the final model, TnT > 600 ng/L was the only variable with significant independent association to 6-months mortality: Hazard Ratio 6.68 (95% confidence interval [CI] 2.24–19.9, p = 0.001). Addition of any other variable, including signs or symptoms and the VARC-2 criterion for MI, did not significantly improve the model

The best sensitivity and specificity of TnT as prediction of 6-months mortality were found at cutoff level > 600 ng/L and were 46% and 90%, respectively. Corresponding values for signs and symptoms were 60% and 79%, and for VARC-2 MI criteria were 33% and 94%, respectively. The area under the receiver-operating characteristic curve for TnT related to 6-months mortality was 0.69 (95% CI: 0.52–0.86; p = 0.027) and for CK-MB it was 0.58 (95% CI: 0.41–0.75; p = 0.36).

## Discussion

Factors related to periprocedural myocardial injury in patients undergoing TAVI have been pointed out in previous studies [6]. Such are the TA approach, number of rapid pacing episodes with the following hypotension, degree of prosthesis oversizing, and coronary embolism. These factors are strictly related in time to the valve implantation procedure. Occurrence of symptoms in sedated patients during this period is difficult to obtain. Signs, such as hypotension, ventricular arrhythmia, temporally ECG changes are regularly resulted by rapid pacing and catheter positioning [7].

The original version of universal definitions of MI recommended an elevation of biomarkers alone for the diagnosis of post-percutaneous coronary intervention (PCI) MI [8]. The requirement for clinical, ECG or angiographic changes for the diagnosis of post-PCI MI was added to the criterion in the revised version [9]. Moussa et al. define post-PCI and post-coronary artery bypass surgery MI as ECG changes, new Q-wave or left bundle branch block, in addition to biomarker elevation [10]. Obviously there is some uncertainty whether postprocedural biomarker elevation alone should define periprocedural MI.

Our data shows that patients with signs or symptoms of MI have significantly higher levels of TnT and CK-MB, but also that 44% and 50% of the patients with TnT > 600 ng/L and CK-MB > 25 μg/L, respectively, had no signs and symptoms at all. Furthermore, there were no significant differences in the levels of biomarkers of MI in patients with and without signs and symptoms when the postprocedural TnT was above 600 ng/L.

Although there was correlation between the postprocedural levels of TnT and CK-MB, the coherency above the cut-off levels of 600 ng/L for TnT and 25 μg/L for CK-MB was defective as 9 of the 16 patients with TnT > 600 ng/L had CK-MB < 25 μg/L. Two patients had serious limb ischemia after TAVI with TF approach resulting in CK-MB elevation without rise in TnT. TnT is influenced by renal function but it is still more specific for myocardial injury than CK-MB [11]. Our data shows no correlation between CK-MB > 25 μg/L and 30-day or 6-months mortality. Similarly Rodes-Cabau et al. found no correlation between CK-MB rise and 30-day mortality after TAVI, however, postprocedural TnT elevation was found as an independent risk factor of cardiac mortality in their study [6].

In the VARC-2 definition of periprocedural MI, the cut off level of troponin is set to 15x the upper reference limit. Rodés-Cabau et al. found a TnT cutoff point > 600 ng/L best determining the occurrence of cardiac death and a TnT cutoff point > 480 ng/L best predicted a decrease in left ventricle ejection fraction following TAVI [6]. These levels correspond well with our findings and they are in accordance with 15x the upper reference limit (30–40 ng/L) for previous troponin analysis methods. In order to detect minor spontaneous MI, the tests for troponins have been made more sensitive in the low ranges and they allow a decrease in upper reference limit [12]. At our laboratory, troponin is analyzed as high sensitive troponin T with an upper reference limit 15 ng/L, which alters the required elevation of TnT to 225 ng/L if the present VARC-2 definition for periprocedural MI is followed. Consequently, changes in upper reference limit due to change to high sensitive assays should not affect the cut-off level for the MI diagnosis at TAVI procedures. Alternatively, a different multiply of the upper reference limit of the high sensitive assays for TnT should be used.

In our study, TnT > 600 ng/L, signs and symptoms, and VARC-2 criteria for periprocedural MI all showed a significant correlation to adverse procedure outcome in terms of 6-months mortality, NT-proBNP elevation, renal impairment, and need of ICU care. TnT > 600 ng/L had the best predictive value of 6-months mortality and in the multivariate Cox regression model it was the only independent significant variable predicting 6-monts mortality.

We found a higher incidence (10%) of periprocedural MI according to the VARC-2 definition than the 1% to 3% reported in other publications, using the same criteria.[13–15] However, our 13% incidence of patients with TnT > 600 ng/L is lower than the 40% reported in uncomplicated TAVI procedures by Rodés-Cabau [6]. The difference in incidence of periprocedural MI in our study compared to others might be a manifestation that the signs and symptoms of MI at TAVI procedures are obscure, difficult to interpret, and not comparable with the clinical manifestation of spontaneous MI.

In accordance with previous studies, we found a higher incidence of myocardial injury and MI according to the VARC-2 criteria in TA patients compared with TF patients, but the difference in occurrence of signs and symptoms did not reach significant level [6].

### Study limitations

The study population is relatively small and although it allows the statistic calculations conducted, the results must be interpreted in the light of the sample size and they must also be verified in larger, multicenter studies. Data on preprocedural CK-MB and TnT were incomplete and we can therefore not exclude the possible occurrence of preprocedural biomarker elevation. If so, the frequency of MI according to the VARC-2 criteria might have been somewhat lower.

## Conclusions

Postprocedural elevation of troponin T after TAVI adequately reflects the periprocedural myocardial injury and it is related to adverse procedure outcome. In fact, TnT > 600 ng/L was the only independent variable predicting 6-months mortality. The signs and symptoms in the VARC-2 criteria add little or nothing to the diagnosis of periprocedural MI. However, signs and symptoms are absent in nearly half of the patients with postprocedural TnT > 600 ng/L. We therefore suggest that periprocedural MI at TAVI should solely be based on levels of biochemical markers of myocardial injury, preferably troponins. A cutoff level of TnT > 600 ng/L on the third day after TAVI procedures seems to be adequate, independently of the local upper reference limit. Such a criterion for periprocedural MI at TAVI would facilitate the unbiased comparison of different patient materials and provide a powerful prognostic variable.

## References

[1] Cribier A, Eltchaninoff H, Bash A, Borenstein N, Tron C, Bauer F, et al Percutaneous transcatheter implantation of an aortic valve prosthesis for calcific aortic stenosis: first human case description. Circulation. 2002;106: 3006–3008. 12473543 10.1161/01.cir.0000047200.36165.b8

[2] Al-Attar N, Himbert D, Descoutures F, Iung B, Raffoul R, Messika-Zeitoun D, et al Transcatheter aortic valve implantation: selection strategy is crucial for outcome. Ann Thorac Surg. 2009;87: 1757–1763. 10.1016/j.athoracsur.2009.03.047 19463591

[3] Lange R, Bleiziffer S, Mazzitelli D, Elhmidi Y, Opitz A, Krane M, et al Improvements in transcatheter aortic valve implantation outcomes in lower surgical risk patients: a glimpse into the future. J Am Coll Cardiol. 2012;59: 280–287. 10.1016/j.jacc.2011.10.868 22196885

[4] Kappetein AP, Head SJ, Genereux P, Piazza N, van Mieghem NM, Blackstone EH, et al Updated standardized endpoint definitions for transcatheter aortic valve implantation: the Valve Academic Research Consortium-2 consensus document. J Thorac Cardiovasc Surg. 2013;145: 6–23. 10.1016/j.jtcvs.2012.09.002 23084102

[5] Appel CF, Hultkvist H, Nylander E, Ahn H, Nielsen NE, Freter W, et al Transcatheter versus surgical treatment for aortic stenosis: patient selection and early outcome. Scand Cardiovasc J. 2012;46: 301–307. 10.3109/14017431.2012.699636 22656069

[6] Rodes-Cabau J, Gutierrez M, Bagur R, De Larochelliere R, Doyle D, Côté M, et al Incidence, predictive factors, and prognostic value of myocardial injury following uncomplicated transcatheter aortic valve implantation. J Am Coll Cardiol. 2011;57: 1988–1999. 10.1016/j.jacc.2010.11.060 21565636

[7] Gutierrez M, Rodes-Cabau J, Bagur R, Doyle D, DeLarochelliere R, Bergeron S, et al Electrocardiographic changes and clinical outcomes after transapical aortic valve implantation. American Heart Journal. 2009;158: 302–308. 10.1016/j.ahj.2009.05.029 19619709

[8] Thygesen K, Alpert JS, White HD, Jaffe AS, Apple FS, Galvani M, et al Universal definition of myocardial infarction. Circulation. 2007;116: 2634–2653. 17951284 10.1161/CIRCULATIONAHA.107.187397

[9] Thygesen K, Alpert JS, Jaffe AS, Simoons ML, Chaitman BR, White HD, et al Third universal definition of myocardial infarction. Circulation. 2012;126: 2020–2035. 10.1161/CIR.0b013e31826e1058 25689940

[10] Moussa ID, Klein LW, Shah B, Mehran R, Mack MJ, Brilakis ES, et al Consideration of a new definition of clinically relevant myocardial infarction after coronary revascularization: an expert consensus document from the Society for Cardiovascular Angiography and Interventions (SCAI). Journal of the American College of Cardiology. 2013;62: 1563–1570. 10.1016/j.jacc.2013.08.720 24135581 PMC3890321

[11] Jaffe AS, Babuin L, Apple FS. Biomarkers in acute cardiac disease: the present and the future. J Am Coll Cardiol. 2006;48: 1–11. 16814641 10.1016/j.jacc.2006.02.056

[12] Thygesen K, Mair J, Katus H, Plebani M, Venge P, Collinson P, et al Recommendations for the use of cardiac troponin measurement in acute cardiac care. Eur Heart J. 2010;31: 2197–2204. 10.1093/eurheartj/ehq251 20685679

[13] Bleiziffer S, Mazzitelli D, Opitz A, Hettich I, Ruge H, Piazza N, et al Beyond the short-term: clinical outcome and valve performance 2 years after transcatheter aortic valve implantation in 227 patients. J Thorac Cardiovasc Surg. 2012;143: 310–317. 10.1016/j.jtcvs.2011.10.060 22137802

[14] Fassa AA, Himbert D, Vahanian A Mechanisms and management of TAVR-related complications. Nature reviews Cardiology. 2013;10: 685–695. 10.1038/nrcardio.2013.156 24101101

[15] Genereux P, Head SJ, Van Mieghem NM, Kodali S, Kirtane AJ, Xu K, et al Clinical outcomes after transcatheter aortic valve replacement using valve academic research consortium definitions: a weighted meta-analysis of 3,519 patients from 16 studies. J Am Coll Cardiol. 2012;59: 2317–2326. 10.1016/j.jacc.2012.02.022 22503058

